# Supplementary figures and images for: Efficacy of vocal fold injection of dedifferentiated fat cells in treating glottis closure insufficiency: Insights from a rat model of recurrent laryngeal nerve resection
Source: PLoS One. 2025 Sep 5;20(9):e0324965. doi: 10.1371/journal.pone.0324965 (PMC12412957; doi:10.1371/journal.pone.0324965)

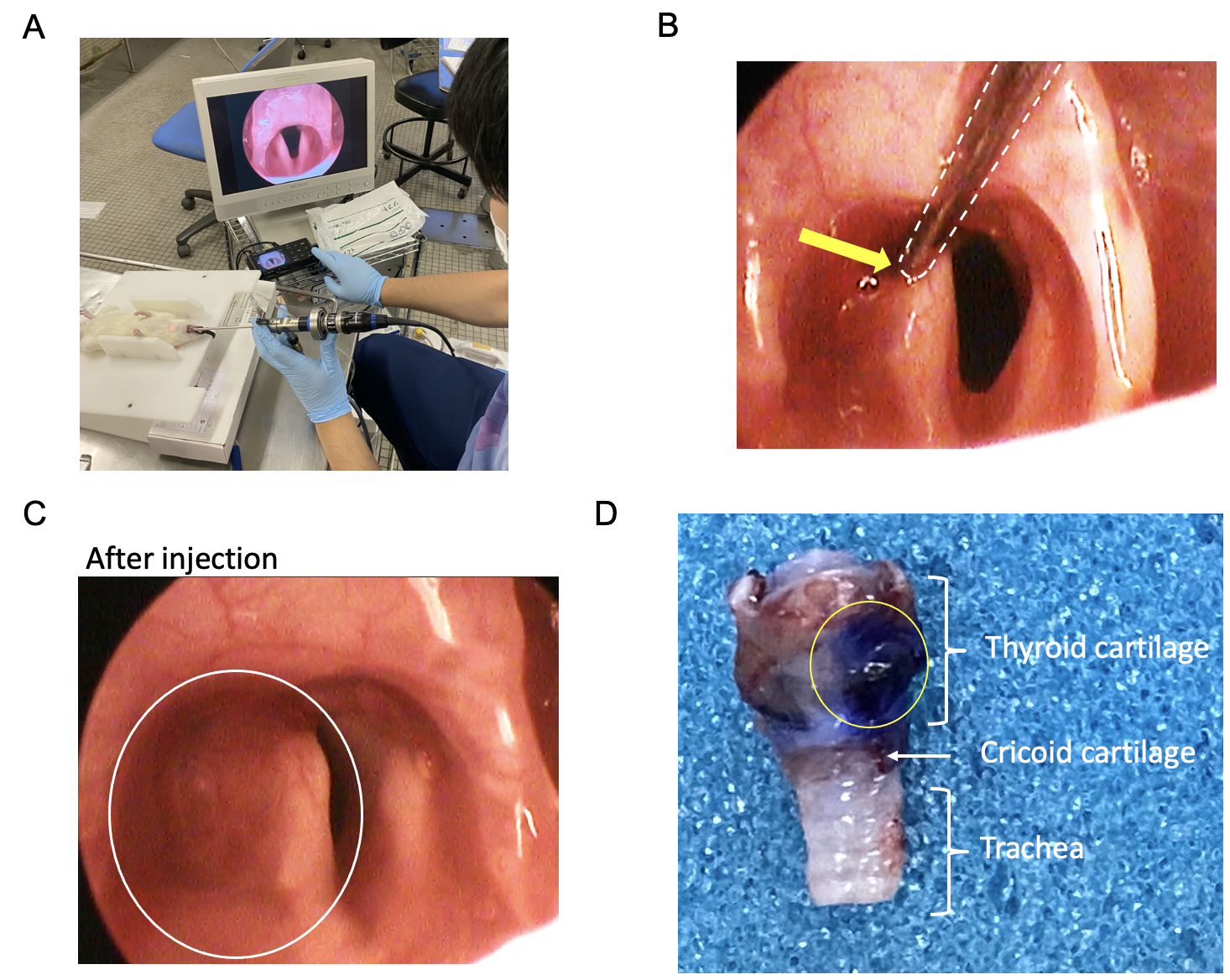

Supplement: S1 Fig — After general anaesthesia was administered to Sprague-Dawley (SD) rats, 10 µl of collagen gel containing pioctanine dye was injected into the vocal fold muscle, using a custom-made injection needle and micro syringe, under the guidance of a rigid endoscope (TrueView II; OLYMPUS). After injection, the rats were euthanized, and the larynges were excised for examination. (A) Endoscopic view of the intralaryngeal vocal fold muscle injection procedure. (B) Rigid endoscopic image during vocal fold injection. Dotted line indicates the injection needle; arrow points to the injection site. (C) Rigid endoscopic image immediately after injection. (D) Appearance of the larynx after laryngectomy. (TIFF) [file pone.0324965.s001.tiff]

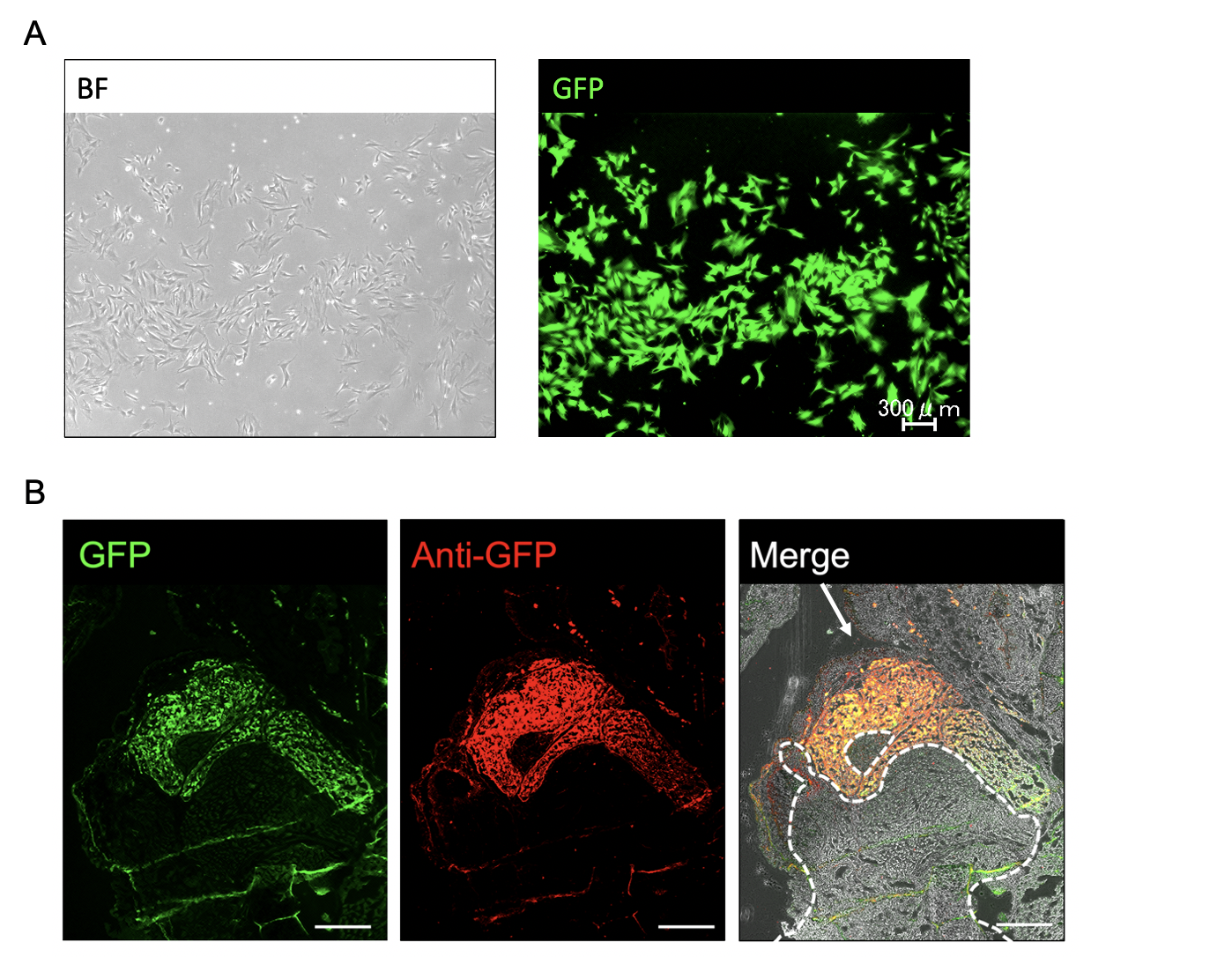

Supplement: S2 Fig — GFP-labelled DFATs mixture (10 µl) was injected endoscopically into the vocal fold muscles of the rats; and immediately afterwards the rats were euthanized and the larynges were excised. Frozen specimens were then prepared for immunohistological evaluation. (A) DFATs during culture. Scale bar = 300 µm. (B) Fluorescence immunostained image of laryngeal tissue post-transplantation. Scale bar = 200 μm. Arrow: injection point; dotted line: muscle area. (TIFF) [file pone.0324965.s002.tiff]

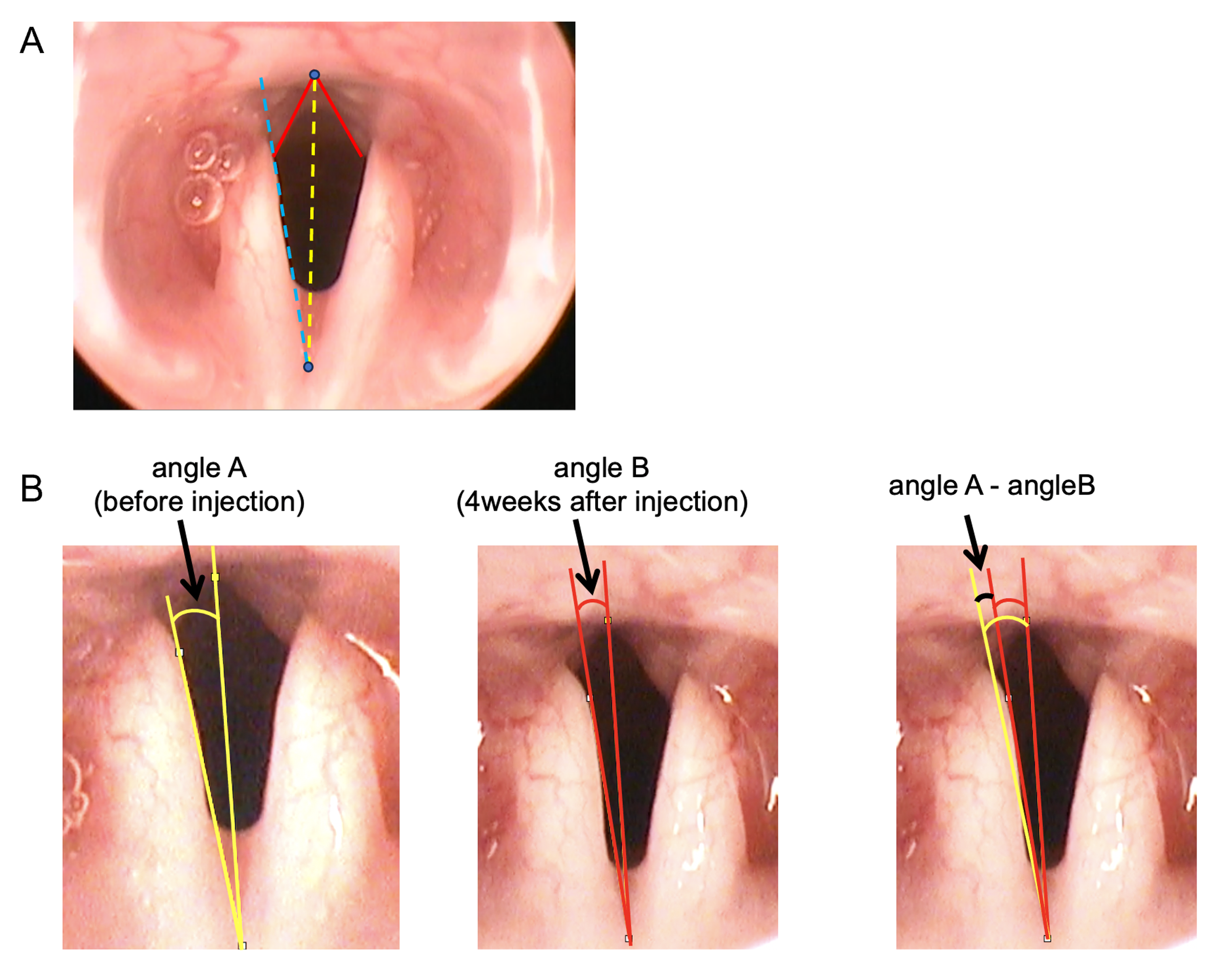

Supplement: S3 Fig — The adduction angle of the arytenoid cartilage was measured using still images extracted from the endoscopic videos, captured at the end of exhalation when the vocal folds were momentarily stationary. (A) The midline of the glottis (yellow dotted line) was determined at the line connecting (1) the intersection point of tangents drawn along the medial edges of the bilateral vocal folds (red lines), and (2) the junction of the bilateral arytenoid cartilages (blue dotted line). (B) The angle formed by the midline of the glottis and a tangent drawn from the junction of the arytenoid cartilages to the left (treated) arytenoid cartilage was measured before injection (angle A) and 4 weeks after injection (angle B), respectively. The adduction angle (angle A – angle B) and the improvement rate of the glottic gap ([angle A – angle B]/angle A) were then calculated. (TIF) [file pone.0324965.s003.tif]

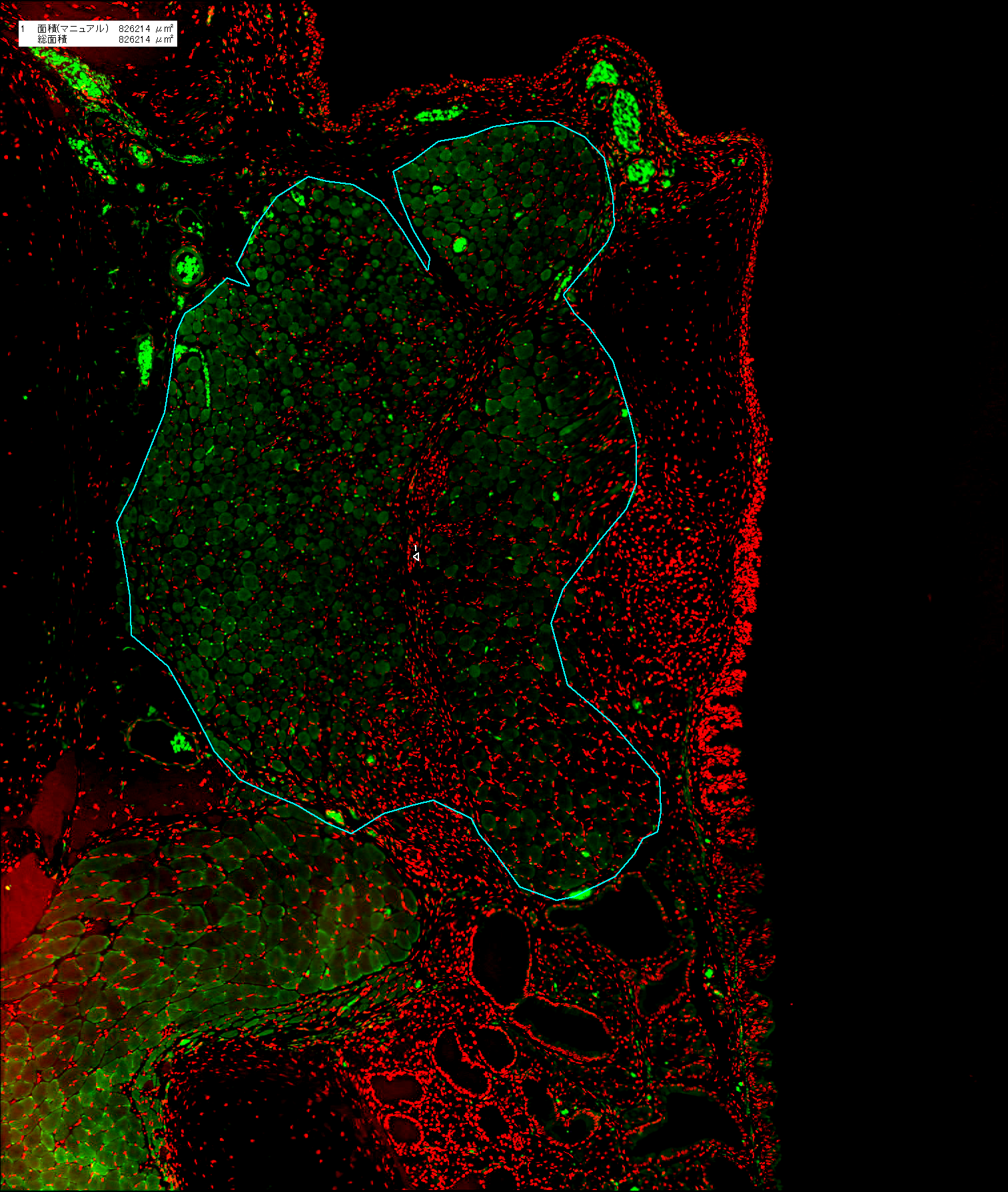

Supplement: S1 File — (ZIP) [file pone.0324965.s004.zip › minimal data set/Fig.4_Ki67-positive cells_image.tif]

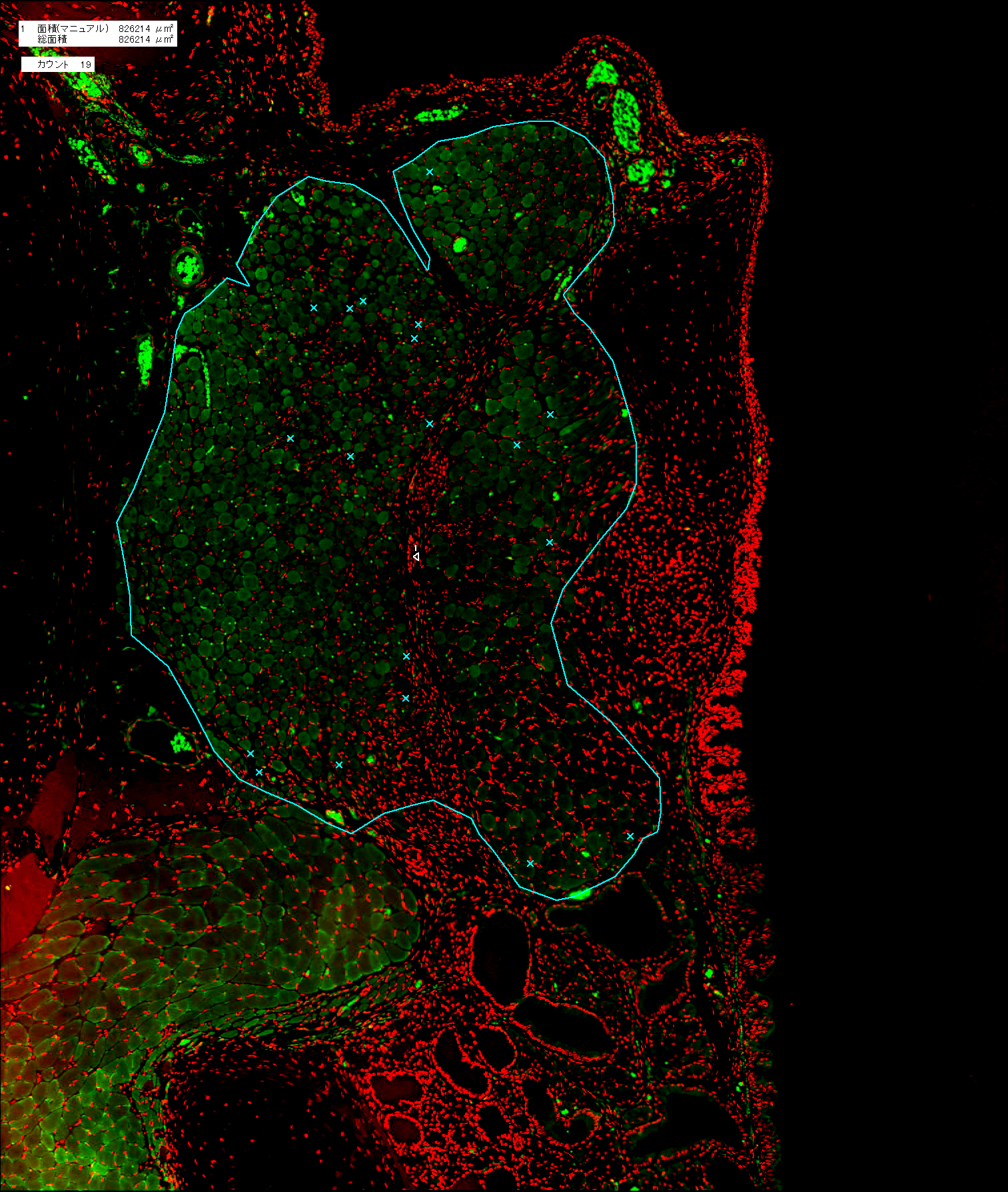

Supplement: S1 File — (ZIP) [file pone.0324965.s004.zip › minimal data set/Fig.4_Ki67-potitive cells_count image.tif]

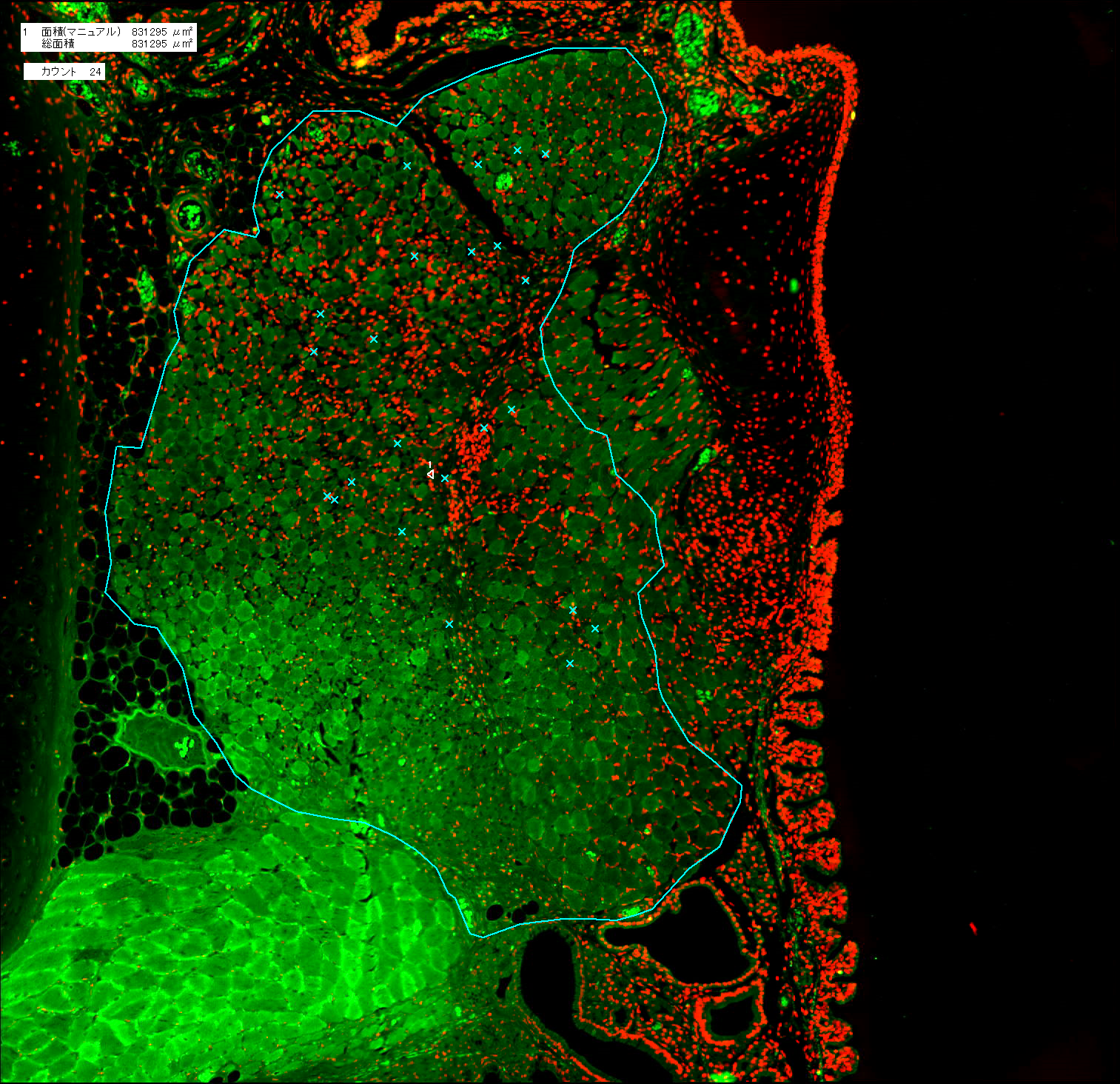

Supplement: S1 File — (ZIP) [file pone.0324965.s004.zip › minimal data set/FIg.5_TUNEL-positive cells_count image.tif]

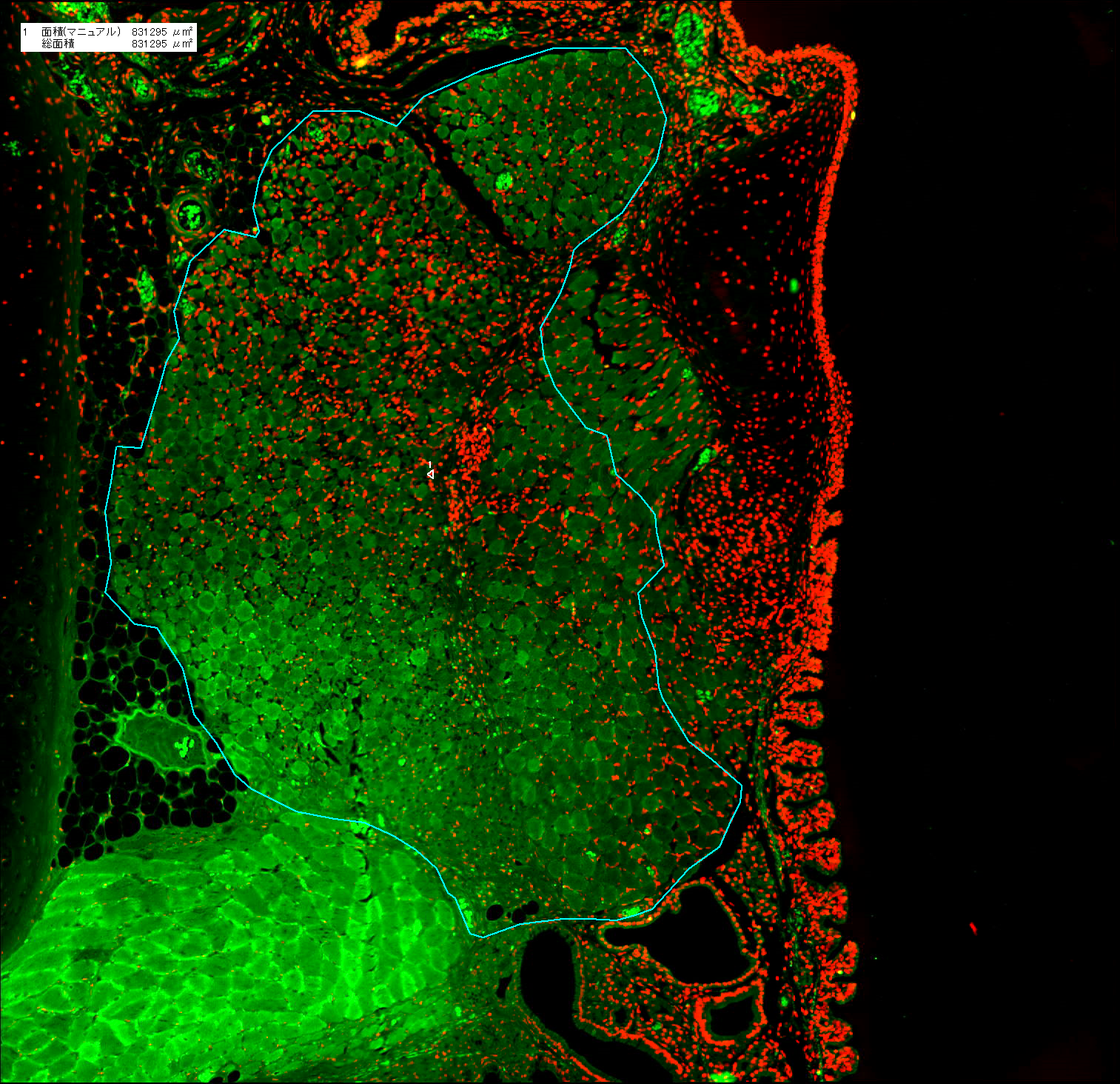

Supplement: S1 File — (ZIP) [file pone.0324965.s004.zip › minimal data set/Fig.5_TUNEL-positive cells_image.tif]
