# Supplementary material for: Efficacy of vocal fold injection of dedifferentiated fat cells in treating glottis closure insufficiency: Insights from a rat model of recurrent laryngeal nerve resection
Source: PLoS One. 2025 Sep 5;20(9):e0324965. doi: 10.1371/journal.pone.0324965 (PMC12412957; doi:10.1371/journal.pone.0324965)
Supplement: S1 File — (ZIP) [file pone.0324965.s004.zip › minimal data set/Read Me.docx]

The minimal data set underlying the findings of this study is provided as follows:

a) The values behind the means, standard deviations, and other statistical measures reported;

b) The values used to construct the graphs;

c) The points extracted from images for analysis

For a) and b), the raw numerical data used for each graph have been provided in Excel file format.

For c), the image data used for analysis are presented as follows:

・Fig. 1: Corresponding data are shown in Fig. A and S3_Fig.

・Fig. 2: Data points are presented in Fig. 2B

・Fig. 3: Data points are shown in Fig. 3B.

・Fig. 4 (Number of Ki-positive cells) and Fig. 5 (Number of TUNEL-positive cells): The images used for analysis are provided as TIFF files.
